# Global commitments and China’s endeavors to promote health and achieve sustainable development goals

**DOI:** 10.1186/s41043-018-0139-z

**Published:** 2018-04-12

**Authors:** Xiaodong Tan, Qian Wu, Haiyan Shao

**Affiliations:** School of Health Sciences, Wuhan University, Wuhan, Hubei China

**Keywords:** Healthy China 2030, Sustainable development goals, Burden of chronic diseases, Health expenditure, Health insurance

## Abstract

**Background:**

With its immense population and as the largest developing country in the world, China has made remarkable achievements in health promotion at a relatively low cost. However, China is still faced with challenges such as changes of disease spectrum, the coming era of an aging society, and the risk of environmental pollution.

**Main text:**

On October 25, 2016, China formally passed the blueprint of “Healthy China 2030,” working towards the national goal of reaching a health standard on par with developed countries by 2030, which was also a response to realize the 2030 United Nations Sustainable Development Goals. “Healthy China 2030” is comprised of 29 chapters that cover five health areas. China is sparing no effort to transfer from being merely the most populous country, to becoming a leading nation in health education. In “Healthy China 2030,” collaborated construction and resource sharing were clearly stated as the core strategy. A shift in concentration towards coordinated development of health-based economy from a previous pursuit of rapid economic growth was also underlined. There are also several major issues, such as severely aging population, the burden of chronic diseases, the insufficiency of health expenditure, and the great demand on health protection, waiting to be dealt with during the implementation process of “Healthy China 2030”.

**Conclusions:**

“Healthy China 2030” is a momentous move to enhance public health, which is also a response to the global commitments. We also need to rethink our approach to reach the living standards and maintain a better environment.

## Background

With its immense population and as the largest developing country in the world, China has made remarkable achievements in health promotion at a relatively low cost [1]. To weigh the health level of countries or regions, Global Burden of Diseases 2015 provided a comprehensive assessment of global life expectancy from 1980 to 2015. Within the last 25 years, global life expectancy at birth increased by 10.2 years, rising from 61.7 to 71.8 years in 2015. The life expectancy of Chinese residents has increased to 76.2 years, 4.4 years higher than the international average [2, 3]. Although such achievements have been made, China is still faced with challenges such as changes of disease spectrum and the coming era of an aging society. As the country’s population has continued to age, the elderly over 60 years old accounted for 17.3% of the total population in 2017 [4]. Furthermore, due to the rapid development of automobile industry, the risk of environmental pollution has become increasingly serious.

## A brief introduction to healthy China 2030

On August 19, 2016, the Chinese government held the “National Health and Healthcare Conference” [5], in which the framework of “Healthy China 2030” was under deliberation. On October 25, 2016, China formally passed the blueprint of “Healthy China 2030”, working towards the national goal of reaching a health standard on par with developed countries by 2030. Another positive response to health promotion was the 9th Global Conference on Health Promotion (9GCHP), co-organized by WHO and the National Health and Family Planning Commission (NHFPC) of the People’s Republic of China, in which health promotion was reiterated as a top-priority political strategy [6]. “Healthy China 2030” was also a response to the 2030 United Nations Sustainable Development Goals (SDGs) [7], in which 17 goals were issued, and “Healthy China 2030” will promote the realization of goal 3 and goal 6—“Ensure healthy lives and promote well-being for all at all ages” and “Ensure access to water and sanitation for all”.

“Healthy China 2030” is comprised of 29 chapters that cover five areas such as public health services, environment management, the medical industry, and food and drug safety. A national health file with such precise indices is rare. The blueprint stipulates several goals to be reached by the target date, as listed in Table 1. There are twelve major indices: five health outcome indices, one healthy lifestyle index, three health service and health insurance indices, two health environment indices, and one health industry index [8]. The goal of increasing Chinese citizens’ average life expectancy is clearly defined as reaching 77.3 years by 2020 and 79 years by 2030. Infant mortality rate is targeted to decline to 9.5 deaths per 1000 live births in 2020 and 6.0 deaths per 1000 live births in 2030. In recent years, recurrent large-scale fog events have aroused widespread social concern. To alleviate this, a notable indicator was detailed in the blueprint and the ratio of superior air quality days in central cities (aiming to reach 76.7% in 2020 and > 80% in 2030) was formulated to control the prominent environmental pollution-related health problems.

**Table 1:** Major indices of “Healthy China 2030” construction

Major indices of Healthy China	Target date		
	2015	2020	2030
Health outcome			
Life expectancy (years)	76.34	77.3	79.0
Infant mortality rate (%)	8.1	7.5	5.0
Under-five mortality rate (%)	10.7	9.5	6.0
Maternal mortality rate (/100,000)	20.1	18.0	12.0
Qualification rate of the national physique determination standard (%)	89.6	90.6	92.2
Health environment			
Ratio of superior air quality days in central cities (%)	76.7	> 80	–
Rate of reaching case III surface water standard (%)	66	> 70	–
Health service and health insurance			
Premature mortality from major chronic diseases (%)	19.1	17.2	12.0
Licensed doctors (assistants)/1000 residential population	2.2	2.5	3.0
Health expenditure personal/national health expenditure (%)	29.3	28	25
Healthy lifestyle			
Residents' health literacy level (%)	10	20	30
Population exercising regularly (billion)	3.6	4.35	5.3
Health industry			
Total scale of health services (trillion yuan)	–	> 8	16

## Significances of “Healthy China 2030”

As the national strategic plan in health for the next 15 years, “Healthy China 2030” is special and significant in several ways.

Firstly, the innovative development concept has Health in All Policies (HiAPs) as its foremost consideration [9]. China is sparing no effort to transform itself from being merely the most populous country into a leading nation in health education and incorporate health education into its national education system. The function of health services is transferring from basic disease treatment to health promotion and health management. A shift in the country’s concentration towards a coordinated development of health-based economy from a previous pursuit of rapid economic growth was also underlined in the plan.

Secondly, to emphasize the significance of health for everyone, collaborated construction and resource sharing are also identified as the core strategic themes of Healthy China. While the government is playing a leading role, the general public should also be mobilized to participate in health maintenance initiatively. The health of the whole society is inseparable from the combined efforts of all members. Citizens must be responsible for their own well-being, encouraged by the concept of “My Health under My Control” by promoting social responsibility, knowledge, and capability [10].

Thirdly, with a rising demand on health, health market will become a new economic growth point. As China has entered a critical period of economic transition, the industrial structure is in the process of readjustment. Firstly, an opportunity in emerging industry like aged care (with China becoming an aging country at an unprecedented level [11]), tourism, fitness, and leisure industry will potentially become the next engine of economic growth. Secondly, people’s shift in demand from disease treatment to health promotion accelerates the burgeoning industry of Chinese medicine services as a unique health economic resource. Policies and measures have already been formulated to expedite the development of Chinese medicine health care services, by encouraging social and economic forces into the traditional Chinese medicine health care institutions and supporting pension institutions in cooperating with traditional medicine institutions [12]. "Healthy China 2030" stressed on combining traditional Chinese medicine with health management and promoting the inheritance of traditional Chinese medicine culture as well.

The last but not the least, there are some other actions of "Healthy China 2030", which may bring about profound significance, such as encouraging the development of commercial health insurance as a supplement for health insurance system and combining Healthy China with new media to stimulate people’s consumption on health. The government tries to provide multiple health options to promote people’s well-being in all aspects.

## Major issues requiring further discussion

“Healthy China 2030” is not simply a major drive to improve the health of the Chinese population. It also plays an integral role in engaging global health governance and implementing the country’s commitment to the SDGs. A severely aging population, the burden of chronic diseases, and multiple health threats posed by environmental pollution are urgent problems shared between China and the rest of the world over the next 15 years, and these issues remain to be further discussed.

As another index of premature mortality from major chronic diseases is expected to fall by 30%, however, a study from CDC in 2017 showed that two thirds of the less developed provinces/municipalities will not reach the target by 2030, for instance Qinghai Province reaching 28.81%, Tibet reaching 25.88%, while the most developed municipalities like Shanghai and Beijing reaching 8.40 and 9.39%, respectively [13]. However, as depicted in Healthy China 2030, premature mortality from major chronic diseases is expected to decline to 17.2% in 2020 and 12.0% in 2030. There is therefore a long way to go before effectively reducing levels of premature mortality from major chronic diseases to the target. The burden of chronic diseases is still severe, the mortality of which accounting for 86.6% of the total death in 2012 [14], and about one third of Chinese adults had hypertension [15]. Pertinent measures, such as improving the health insurance system and establishing the intervention plan, must be taken, especially in economically backward regions.

Health expenditure finance can hardly meet the increasingly public medical need. Data from The World Bank showed that the average global health expenditure reaching 9.9% of GDP in 2014, with health expenditure accounting for 17.1% in the USA, 11.9% in Sweden, 11.3%in Germany, and 10.2% in Japan, while China’s health costs accounted for 5.5% of its GDP in 2014, trailed behind the developed countries significantly [1]. An adequate health policy and an increased investment are vital to change the status quo and fundamentally accelerate the transformation of government functions.

What is more, despite the great achievements in social primary health insurance, there are still some shortcomings such as irrational individual expenses and not including the specific diseases in health insurance, thus, a more rational and extensive covered health insurance is needed. In 2017, the government implemented some health-insurance changes, for example, increasing the aid-the-poor funds according to the proportion of supportive funds and expanding insurance coverage. With a review of strategies in the developed countries [16], improvements in national health will secure economic prosperity and ensure its own health and human security.

## Conclusions

“Healthy China 2030” is a momentous endeavor to enhance public health and a response to the global commitments. Setting the long-term targets, combined with promoting HiAPs and collaborated construction and resource sharing on arising health issues, China explores a remarkable way to tackle these issues, such as rapid aging, growth of NCDs, and an inflation of health demands. However, there are still some major issues, including the rationality of indices and the coverage of indices for certain health-related issues, waiting to be further improved in the implementation procession. It is imperative that we rethink approaches to reach the required living standards and maintain a better environment.

## Abbreviations

9GCHP: The 9th Global Conference on Health Promotion
HiAPs: Health in All Policies
NHFPC: The National Health and Family Planning Commission
SDGs: The 2030 United Nations Sustainable Development goals
